# Quadruple dislocation fracture: concurrent glenoid, greater tuberosity, coracoid process, and acromion fractures following anterior shoulder dislocation

**DOI:** 10.1016/j.xrrt.2024.12.015

**Published:** 2025-01-29

**Authors:** Arashk Ghasroddashti, Colm Guyn, Joseph Bergman

**Affiliations:** a School of Medicine, Faculty of Health Sciences, Queen's University, Kingston, Ontario, Canada; b Faculty of Medicine and Dentistry, University of Alberta, Edmonton, Alberta, Canada; c Division of Orthopedic Surgery, Department of Surgery, University of Alberta, Edmonton, Alberta, Canada; d Western Upper Limb Facility, Sturgeon Community Hospital, St. Albert, Alberta, Canada

**Keywords:** Glenohumeral dislocation, Shoulder dislocation, Anterior shoulder dislocation, Hill-achs, Bankart, Coracoid process fracture

Anterior glenohumeral joint dislocations are estimated to represent approximately 50% of clinically encountered joint dislocations.^3^ The mechanism of injury typically involves forceful external rotation and abduction of the arm during a traumatic event, such as a fall or motor vehicle collision.^3^ Glenohumeral dislocations often present with secondary injuries, ranging from labral tears and ligamentous sprains to osseous lesions.^16^ The most common secondary osseous injuries are bony Bankart and Hill–Sachs lesions, reported in up to 44% and 93% of traumatic anterior glenohumeral dislocations, respectively.^1^ In contrast, fractures of the coracoid process and acromion are rare in this context, with coracoid fractures reported in no more than 2% of cases and reports of acromion fractures limited to case reports and small case series.^9^^,^^13^ Nevertheless, there have been sparse reports of “triple dislocation fractures” of the glenoid, greater tuberosity (GT), and coracoid process in the literature.^4^^,^^13^^,^^15^ In this case report, we present the first case, to our knowledge, of a quadruple dislocation fracture: an anterior shoulder dislocation with concurrent fractures of the glenoid, GT, coracoid process, and acromial tip. We discuss patient risk factors for this injury pattern, a proposed mechanism for the interrelated fractures, and an approach to surgical fixation.

## Case description

**Figure 1:**
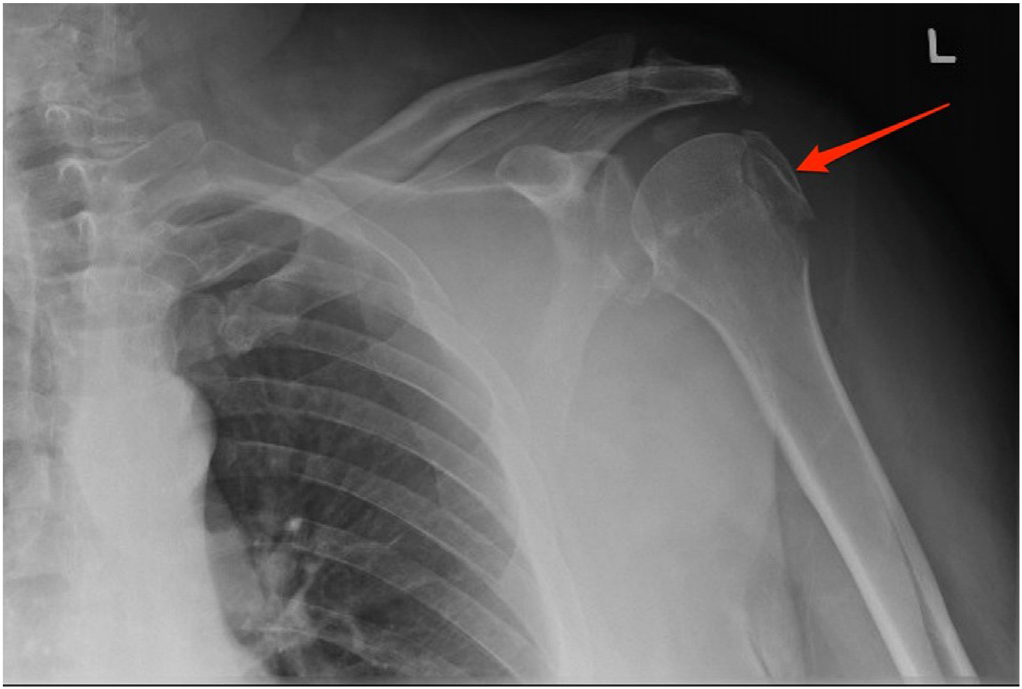
Radiography from initial emergency room visit. *Red arrow* indicates acute greater tuberosity fracture.

A patient in his mid 50s with a body mass index (BMI) of 40 kg/m^2^ and alcohol use history in excess of 28 drinks per week sustained multiple left shoulder fractures following a ground-level fall. For 4 days postinjury, the patient continued to work as a security guard without incident. However, on the fifth day, he noticed increasing discomfort with shoulder use and reported to the emergency room. Initial radiography was reported to demonstrate an acute displaced fracture of the GT with congruent glenohumeral and acromioclavicular joints (Fig. 1).

**Figure 2:**
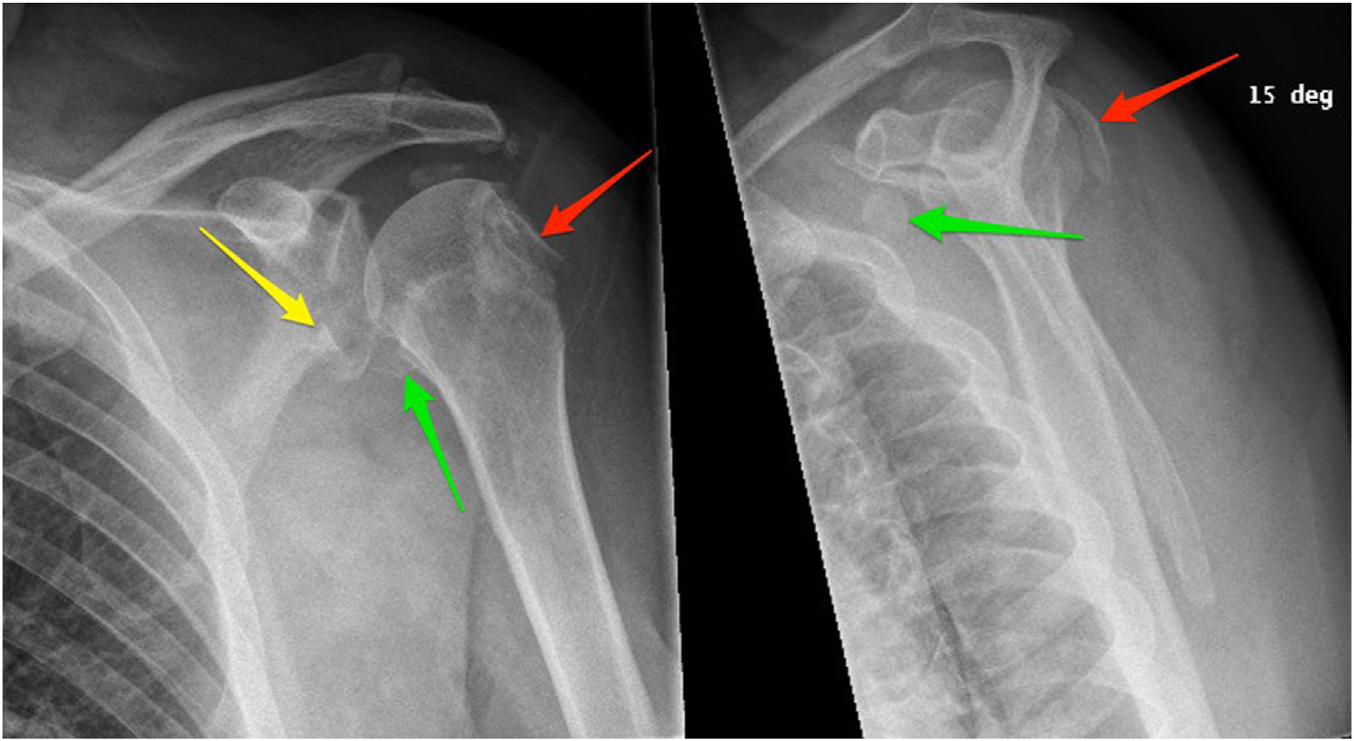
Radiography (2 views) from orthopedic surgery consultation. *Red arrows* indicate Hill–Sachs deformity, *yellow arrow* indicates bony Bankart lesion, and *green arrows* indicate coracoid avulsion.

**Figure 3:**
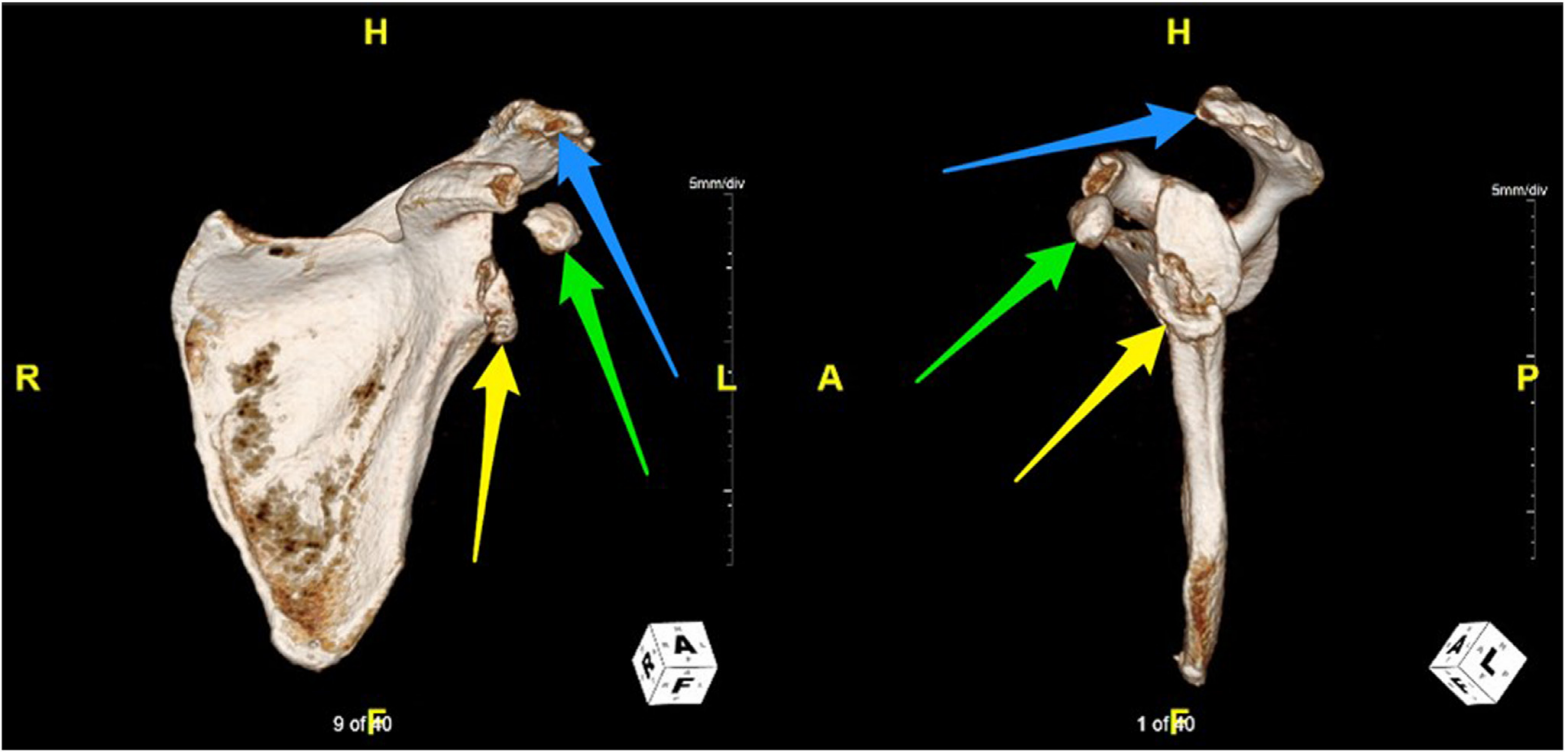
Three-dimensional computed tomography reconstruction (2 views) following orthopedic surgery consultation. *Yellow arrows* indicate bony Bankart lesion, *green arrows* indicate coracoid avulsion, and *blue arrows* indicate likely site of acromial tip avulsion.

During orthopedic surgery consultation 2 weeks postinjury, another x-ray was obtained and revealed a bony Bankart lesion with anteroinferior displacement, a large Hill–Sachs deformity, and a displaced fracture of the coracoid process (Fig. 2). Owing to fracture complexity on x-ray, a computed tomography (CT) scan was obtained and confirmed previous findings (Fig. 3). Evidence of moderate to severe acromioclavicular joint space narrowing and likely avulsion fracture of the acromial tip was also found. Interestingly, the glenohumeral joint was congruent at the time of imaging and there was no evidence on history of past or present dislocation. Open reduction and internal fixation of all 4 fractures was planned. The patient was placed in a cuff and collar sling until surgery.

**Figure 4:**
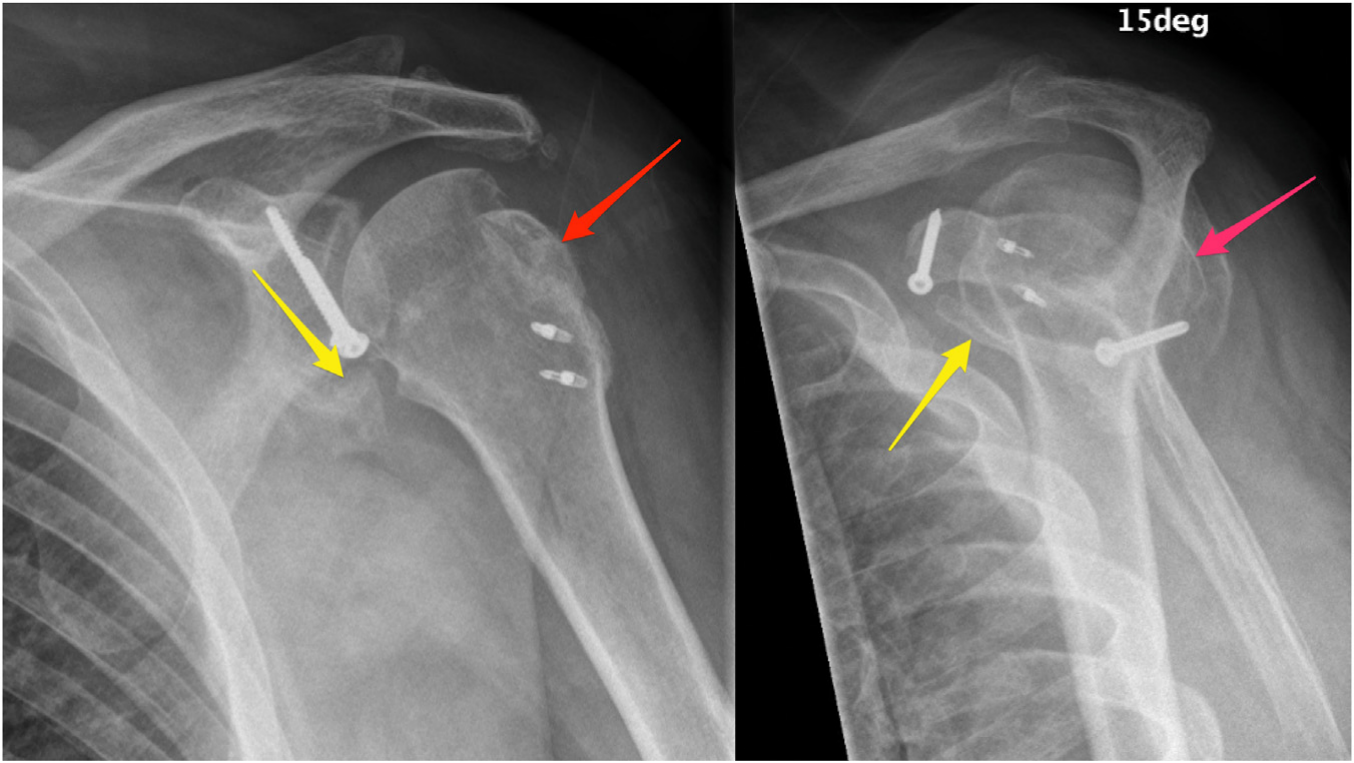
Radiography (2 views) from orthopedic surgery follow-up at 6 weeks postoperatively. *Red arrow* indicates greater tuberosity height loss and *yellow arrow* indicates minimal displacement of inferior glenoid fragment.

Three weeks postinjury, the patient underwent surgery under general anesthesia in the beach chair position. The coracoid tip fracture was first exposed through the deltopectoral interval. The conjoined tendon led up to it and was found intact with the coracoacromial ligament (CAL). Further dissection was performed to the acromial tip fragment. This fragment appeared to be avulsed due to CAL tension but did not involve deltoid. It was decided that excision of the small acromial fragment would minimize the risk of symptomatic nonunion. The intact CAL was sutured back into the area of the defect. Turning to the glenoid fracture next, anteroinferior fragments were left together with only 1 being large enough to accept a 3.5 mm cannulated screw (Fig. 4). This allowed imbrication of the anteroinferior capsule in an open fashion to stabilize the area. Next, a large posterior GT fragment was visualized, removed, and saved. Smaller fragments were captured using 4 loops of braided suture, which was placed through the rotator cuff adjacent to each fragment. Given the significant bone loss from the Hill–Sachs lesion, the rotator cuff was advanced into the defect for joint stabilization. The previously saved GT fragment was then advanced into the defect using 2 stainless steel suture anchors (Fig. 4). The tails of the sutures from this repair construct were used to tie the superior rotator cuff into the Hill–Sachs lesion. Lastly, attention was turned back to the coracoid tip. It was secured with a screw and a hole was drilled through the tip and coracoid shaft, exiting posteromedial to the coracoid angle. This achieved sufficient bony compression. Postoperatively, the shoulder was immobilized using a standard shoulder sling.

The patient returned for follow-up visits at 2 weeks, 6 weeks, 4½ months, and 8½ months postoperatively. At 6 weeks, fractures appeared to be healing well, and, despite some stiffness, pain was dramatically diminished. Some loss of GT height and minimal displacement of the inferior glenoid fragment were noted on radiography (Fig. 4). At 8½ months, pain was resolved, range of motion was satisfactory on examination, and the patient reported adequate functional status. Radiography demonstrated no adverse interval changes, with stable hardware and reductions, as well as fibrous union of the coracoid process fracture.

## Discussion

This case is the first quadruple dislocation fracture reported to our knowledge. It highlights several important considerations when assessing and managing patients with shoulder injuries. First, the initial presentation and injury mechanism of this patient was not characteristic of such a complex and extensive pattern of fractures. The reported trauma was not conventionally high energy and there was minimal pain and loss of function thereafter. Considering patient variability in patient risk factors, pain tolerance, and functional status are crucial, as fractures requiring surgical management can present without significant initial symptoms.

Important patient risk factors were also involved in this case. The patient’s high BMI and significant chronic alcohol use likely contributed to the extent of injury. Patients with elevated BMI tend to experience greater forces during trauma due to the additional mass involved.^5^ It is thus plausible that patients with higher BMI are more likely to sustain extensive injuries even in less severe mechanisms of injury. With respect to alcohol use, chronic consumption is associated with reduced bone density, increasing the likelihood of more severe fractures in trauma.^10^ The pattern of injury in this case is consistent with an anteroinferior glenohumeral dislocation complicated by alcohol-related osteoporotic changes. Although it is not uncommon for bony Bankart and Hill–Sachs lesions to co-occur in anteroinferior dislocations, the fracture of the coracoid process here was likely secondary to either anterior humeral head translation and impact or severe contraction of the pectoralis minor and conjoined tendon in the context of osteoporotic changes.^4^^,^^6^^,^^11^ In either case, avulsion of the acromial tip likely resulted from subsequent increased tension along the CAL. The patient risk factors potentiating these mechanisms underscore the importance of obtaining a complete history in the assessment of suspected traumatic fractures.

CT scans are also recommended to avoid overlooking concurrent injuries in the context of shoulder dislocation.^2^ In this case, initial radiographs did not reveal fractures of the acromion, coracoid, or glenoid. CT helped identify these inconspicuous fractures, which is important as pain and shoulder instability following the treatment of anterior shoulder dislocations are frequently attributed to undiagnosed or untreated osseous defects.^2^^,^^13^ In addition, CT data can help accurately assess fracture displacement, which is integral in determining the need for operative vs. conservative management.^13^ Should operative management be deemed appropriate, 3D reconstructions from CT can also aid in surgical planning.

The questions of operative vs. conservative management of fractures in shoulder dislocation are important to consider. We made the decision to manage this patient’s injury operatively for a number of reasons. First, surgical repair of anterior shoulder dislocations has been reported to improve post-treatment shoulder stability compared to conservative management with recurrence rates of 9% and 62%, respectively.^8^ Second, although the management of coracoid process fractures varies based on their type and severity, operative treatment is generally necessary when concurrent fractures exist.^12^ Lastly, better outcomes have been reported with operative treatment in cases involving large or displaced lesions.^7^^,^^13^ In this patient, the displacement of the glenoid, coracoid process, and GT fragments were best managed surgically. This was done in an effort to optimize long-term glenohumeral joint stability, reduce the likelihood of chronic pain, and delay the development of glenohumeral osteoarthritis.^13^ In cases of similar (typically isolated) fractures with minimal displacement, conservative treatment has also been shown to improve pain and patient satisfaction without recurrent instability.^11^^,^^14^^,^^15^

## Conclusion

There is a large degree of variability across patients in pain perception and functional impairment following traumatic injury to the shoulder. This, along with patient risk factors such as obesity and chronic alcohol consumption, should be considered when estimating the potential extent and severity of traumatic fractures. The appropriate use of high-resolution imaging, such as CT, is recommended as an adjunct to simple x-rays in the assessment of traumatic shoulder dislocation. This imaging modality may help identify additional, inconspicuous fractures and determine the need for surgical management. In cases of concurrent fractures involving the coracoid process, surgical management is recommended to achieve optimal joint stability and pain control.

## Disclaimers:

Funding: No funding was disclosed by the authors.

Conflicts of interest: The authors, their immediate families, and any research foundations with which they are affiliated have not received any financial payments or other benefits from any commercial entity related to the subject of this article.

Patient consent: Obtained.
